# Vaccines in cardiology, an underutilized strategy to reduce the residual cardiovascular risk

**DOI:** 10.47487/apcyccv.v5i1.349

**Published:** 2024-03-19

**Authors:** Sebastián García-Zamora, Laura Pulido

**Affiliations:** 1 Unidad Coronaria del Sanatorio Delta, Rosario, Argentina. Unidad Coronaria del Sanatorio Delta Rosario Argentina; 2 Facultad de Medicina, Universidad Nacional de Rosario (UNR). Universidad Nacional de Rosario Facultad de Medicina Universidad Nacional de Rosario (UNR) Argentina; 3 Servicio de Neumonología del Hospital Italiano de Rosario, Rosario, Argentina. Servicio de Neumonología Hospital Italiano de Rosario Rosario Argentina; 4 Facultad de Medicina, Instituto Universitario Italiano de Rosario (IUNIR). Instituto Universitario Italiano de Rosario Facultad de Medicina Instituto Universitario Italiano de Rosario (IUNIR) Argentina

**Keywords:** Vaccines, Influenza, Pneumonia, Pneumococcal, Herpes Zoster, COVID-19, Respiratory Syncytial Virus, Human, Prevention

## Abstract

Cardiovascular diseases stand as the leading cause of mortality among adults globally. For decades, comprehensive evidence has underscored the correlation between infections, particularly those involving the respiratory system, and an elevated risk of cardiovascular and cerebrovascular events, as well as all-cause mortality. The mechanisms through which infections heighten cardiovascular events are intricate, encompassing immune system activation, systemic inflammation, hypercoagulable states, sympathetic system activation, and increased myocardial oxygen demand. Respiratory infections further contribute hypoxemia to this complex interplay. These mechanisms intertwine, giving rise to endothelial dysfunction, plaque ruptures, myocardial depression, and heart failure. They can either instigate de novo cardiovascular events or exacerbate pre-existing conditions. Compelling evidence supports the safety of influenza, pneumococcal, herpes zoster, COVID-19 and respiratory syncytial virus vaccines in individuals with cardiovascular risk factors or established cardiovascular disease. Notably, the influenza vaccine has demonstrated safety even when administered during the acute phase of a myocardial infarction in individuals undergoing angioplasty. Beyond safety, these vaccinations significantly reduce the incidence of cardiovascular events in individuals with an augmented cardiovascular risk. Nevertheless, vaccination rates remain markedly suboptimal. This manuscript delves into the intricate relationship between infections and cardiovascular events. Additionally, we highlight the role of vaccinations as a tool to mitigate these occurrences and reduce residual cardiovascular risk. Finally, we emphasize the imperative need to optimize vaccination rates among individuals with heart diseases.

## Introduction

Cardiovascular diseases are currently the leading cause of death worldwide, both in developed and developing countries ^1^^,^^2^. Latin America is no exception, and available data suggest that cardiovascular diseases result in nearly double the number of deaths compared to all neoplasms combined. The shift from infectious to non-communicable diseases has been termed “epidemiological transition”, intensifying efforts to reduce the burden of these diseases ^3^.

Thus, despite advances in the treatment of individuals in primary and secondary prevention, a significant number of them continue to experience major cardiovascular events or death. The occurrence of cardiovascular events, despite receiving appropriate preventive treatment according to the clinical context, has been termed “residual cardiovascular risk” and is associated with the presence of various inflammatory states, including infections ^4^. In this review, we will analyze the role of infections as triggers for cardiovascular events and the role of immunizations in preventing the occurrence of these events.

## Infections and cardiovascular events

**Figure 1:**
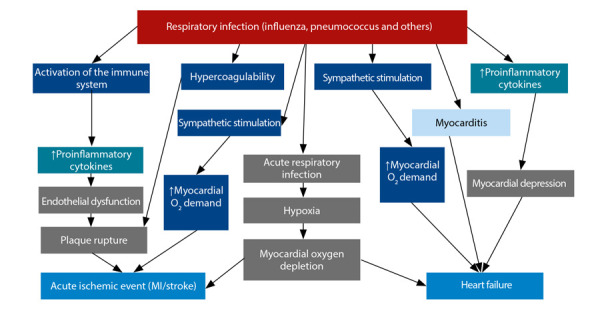
Schematic representation of the relationship between infections and cardiovascular events: it is observed that different mechanisms intervene to trigger an event, and at the same time, similar mechanisms can precipitate different events, according to the individual susceptibility of each subject.

For several decades, it has been documented that acute infections are associated with a transient increase in cardiovascular and cerebrovascular events ^2^^,^^5^^,^^6^. The mechanisms by which these phenomena occur are varied, and include the activation of the immune system, resulting in systemic inflammation, generation of a hypercoagulable state, activation of the sympathetic system, and increased myocardial oxygen demand ^2^ (Figure 1). These interrelated mechanisms lead to endothelial dysfunction, plaque events, and/or myocardial depression and heart failure. Additionally, in some cases, direct myocardial damage (myocarditis) occurs, and in respiratory infections, hypoxaemia may also co-exist with a consequent reduction in oxygen supply to the tissues ^2^.

Regardless of the underlying mechanisms, infections precipitate vascular events, both *de novo*, in people with no previous history, and acute events exacerbating the underlying conditions of those with pre-existing pathologies ^7^. This led to explore the use of antimicrobials in patients with cardiovascular disease to prevent new events. However, a recent systematic review and meta-analysis including 38 clinical trials with 26,638 participants concluded that there is no evidence to support this use for cardiovascular prevention ^8^. In fact, this review suggests that the use of macrolides and quinolones in cardiovascular prevention could be harmful.

On the other hand, vaccines are strategies with proven efficacy in reducing infectious diseases, especially their most severe forms. As a result, the role of immunizations in reducing cardiovascular events has garnered interest in recent years ^2^.

## Influenza vaccine

There are four types of influenza viruses: A, B, C, and D; of these, only influenza A and B viruses have clinical relevance in humans. Influenza A viruses are subdivided based on two surface proteins (H and N), with over 130 subtypes. Influenza B viruses are subdivided into two lineages: Victoria and Yamagata, with multiple subgroups (formally termed “subclades”). Different influenza viruses typically cause annual epidemics, usually during the winter season (between April and October in the southern hemisphere), although viral circulation occurs throughout the year. For years, it has been documented that concurrently with the increase in influenza cases (“peaks”) during the winter months, there is also an exponential increase in cardiovascular events, mainly acute myocardial infarction and stroke ^6^^,^^9^^,^^10^. Although vaccination is an effective strategy to prevent severe forms and complications of influenza, vaccination rates are suboptimal in several regions of the world ^11^. In Latin America, some studies show that more than half of the individuals eligible for the influenza vaccine remain unvaccinated ^12^^,^^13^.

### Reduction of cardiovascular events with influenza vaccine

Due to the relationship between infections, inflammation, and cardiovascular events, several studies have evaluated the role of the influenza vaccine in reducing these events ^5^^,^^6^^,^^8^^,^^14^. One of the pioneering studies was the FLUVACS trial conducted in Argentina, which was a randomized, single-center study. It observed that, after 1 year of follow-up, patients vaccinated after a coronary event or planned revascularization had lower mortality and cardiovascular events ^14^. These observations were confirmed by a meta-analysis of clinical trials, which found that influenza vaccination resulted in a 36% reduction in major cardiovascular events (relative risk [RR] = 0.64, 95% confidence interval [CI]: 0.48-0.86; p=0.003) in individuals at high cardiovascular risk ^15^.

A new systematic review and meta-analysis was published in 2021, which included not only clinical trials but also observational studies ^16^. The authors found that influenza vaccination in patients at high cardiovascular risk or with established cardiovascular disease reduced overall mortality (RR = 0.75, 95% CI: 0.60-0.93; p=0.001), cardiovascular mortality (RR = 0.82, 95% CI: 0.80-0.84; p<0.001), and major cardiovascular events (RR = 0.87, 95% CI: 0.80-0.94; p<0.001) ^16^.

In the same year, the results of the IAMI clinical trial were reported, a randomized, double-blind study which enrolled participants during the acute phase of acute coronary syndrome or high-risk angioplasty. Participants were vaccinated between 24 hours before and 48 hours after the hemodynamic procedure ^17^. The group that received the influenza vaccine experienced lower overall mortality (hazard ratio [HR] = 0.59, 95% CI: 0.39-0.89; p=0.001) and cardiovascular mortality (HR = 0.59, 95% CI: 0.39-0.90; p=0.014) at 12 months of follow-up, without an increase in total adverse events (AE) or serious AEs, despite antiplatelet and anticoagulant therapy ^17^.

### Groups at risk and populations that should be vaccinated

The groups at highest risk for influenza complications are:

- The extremes of life: people aged 65 years and older, and children aged 6 months to 5 years, even in the absence of comorbidities, are more likely to suffer from severe forms of the disease.
- People aged 5 to 64 years, but with risk factors or comorbidities (Table 1).

**Table 1:** Summary of comorbidities and conditions that increase the risk of severe forms and complications of influenza infection, from an eminently cardiologic perspective (adapted from references 2 and 18). Persons with these conditions are indicated for annual influenza vaccination

Heart diseases
- Heart failure*, coronary artery disease with or without revascularization, valve replacement, moderate or severe valve disease, pulmonary hypertension, heart transplantation. - Congenital heart diseases of any severity.
Common comorbidities in cardiology
- Diabetes of any type, chronic respiratory diseases (COPD and asthma of any severity), pregnant women in any trimester of gestation and postpartum women until discharge from maternity (maximum 10 days), obese individuals with a body mass index ≥40 kg/m2, chronic kidney disease on dialysis or with expectations of initiating dialysis in the next six months, nephrotic syndrome, chronic liver disease (including cirrhosis), solid organ neoplasms under treatment, and transplant recipients of any organ.
- Any person working in healthcare institutions
Others
- Various entities: diaphragmatic hernia, individuals with tracheostomies, severe malnutrition, functional or anatomical asplenia (including sickle cell anemia), developmental delay in individuals under 18 years old, neuromuscular diseases with respiratory involvement, and severe congenital malformations. Additionally, household contacts of oncohematological patients or premature infants weighing less than 1500 g. - Congenital or acquired immunodeficiencies (both oncohematological and non-oncohematological), infection by the human immunodeficiency virus (HIV), use of immunosuppressive medication (such as methotrexate, azathioprine or biologic drugs), or use of corticosteroids in high doses for more than 14 days. Chronic treatment with acetylsalicylic acid in children under 18 years of age.

*Regardless of ejection fraction.

COPD: chronic obstructive pulmonary disease.

However, since influenza vaccines are highly safe interventions, the United States, following the H1N1 influenza epidemic, simplified its vaccination schedule, urging that all persons older than 6 months be vaccinated annually ^18^. This strategy has the additional benefit of reducing viral circulation ^19^. In other countries, primarily due to cost and accessibility issues, recommendations focus on prioritizing the most vulnerable populations ^2^^,^^19^.

In simplified terms, all cardiovascular diseases constitute a risk factor that requires annual influenza vaccination regardless of age, except for isolated hypertension ^2^^,^^18^. At the same time, it is worth emphasizing that all healthcare workers should be vaccinated annually.

### Influenza vaccination schedule

Influenza viruses have a high capacity to modify the antigenic components of their surface structure. Thus, the strains circulating annually change, and sometimes even more than one strain circulates in the same season. This explains the need for annual influenza vaccination ^2^^,^^18^^,^^19^.

Traditionally, it has been suggested that the vaccine should be administered at the end of summer or early autumn (between March and April in the southern hemisphere), as seroprotection is achieved 2-3 weeks after vaccination, thus generating an adequate antibody levels prior to the onset of months with high viral circulation. However, it should be noted that viral circulation occurs throughout the year, particularly in tropical climates ^20^. Additionally, there are often early or delayed increases in influenza cases, or even multiple “peaks” in a year ^20^. Because of this, and given the reduction in major cardiovascular events and death with vaccination, the best strategy is to administer the vaccine whenever contact with a high-risk person occurs, if they have not been vaccinated the previous year, or during the current season ^17^. Furthermore, in areas with marked seasonality, it is reasonable to administer a new dose of influenza vaccine prior to the onset of high viral circulation if the last dose of the vaccine was received more than 6 months ago.

An aspect worth highlighting is that, despite variations in viral strains, influenza vaccines have proven to be effective even when there is not complete correlation with circulating strains ^21^.

### Available vaccines and their implications

Globally, influenza vaccines can be trivalent (containing two different strains of influenza A and one lineage of influenza B virus) or quadrivalent (containing two strains of influenza A and two lineages of influenza B) ^18^^,^^19^. Over a decade ago, it was documented that older people have a decreased immune response to various pathogens, a phenomenon termed “immunosenescence” ^2^^,^^22^. More recently, it has been observed that, in parallel, older individuals experience a state of persistent inflammation, known as inflammaging, which also negatively influences the immune response.

To try to compensate for this reduced immune response to both pathogens and vaccination, enhanced influenza vaccines have been developed: adjuvanted vaccines (using MF59 adjuvant, an oil-in-water emulsion) and high-dose vaccines (containing 60 μg of each viral antigen, rather than 15 μg). Enhanced vaccines have been shown to be superior to standard vaccines, both in generating antibodies and in preventing hospitalizations ^19^^,^^23^^-^^25^, making them the recommended strategies in people aged 65 years or older, or in higher-risk populations ^18^^,^^19^ (Table 1). So far, all improved vaccines have demonstrated similar benefits in reducing clinically significant events ^18^^,^^25^^,^^26^.

### Practical aspects of influenza vaccination

- It can be administered simultaneously with other vaccines, but should be given at different sites.
- In anticoagulated individuals, it should be administered in the deltoid region; there is no benefit to using the intramuscular or deep subcutaneous route, and the preferred route should be the one with which the operator is most experienced ^2^. Prolonged compression (approximately two minutes) should be applied to ensure hemostasis.
- In anticoagulated individuals using vitamin K antagonists, it should be confirmed that they are within the therapeutic range, ideally with an International normalized ratio (INR) <2.5. For those using direct oral anticoagulants, one dose can be omitted ^2^.
- It is an inactivated (killed) vaccine, meaning that the viruses do not replicate in the body. Therefore, it can be administered to immunocompromised patients.
- Mild conditions such as colds, rhinitis, diarrhoea or the use of antibiotics are not a contraindication to receiving the vaccine.
- Only 0.2% of adults and 1.3% of children are allergic to egg ^2^^,^^18^, and there is one case of anaphylaxis for every million vaccine doses administered ^27^. Therefore, most people can be vaccinated, even with a documented egg allergy ^(^^2^^,^^18^.
- Contraindications are rare and include a history of anaphylactic reaction to the vaccine or any of its components, or a history of Guillain-Barré syndrome within six weeks of vaccination (having ruled out other aetiologies of Guillain-Barré syndrome).

## Pneumococcal vaccine

Pneumococcal infections are the leading cause of death from vaccine-preventable diseases worldwide ^28^. *Streptococcus pneumoniae* (pneumococcus) is responsible for multiple infectious conditions, in addition to pneumonia, other serious conditions such as meningitis or endocarditis ^2^. Approximately one out of every four community-acquired pneumonias is caused by pneumococcus. Although there is specific antibiotic treatment available, its lethality has not changed in recent decades ^19^.

This disease has a bimodal distribution, affecting mainly young children due to the immaturity of their immune system, and older adults due to immunosenescence ^2^^,^^19^^,^^22^. In addition to pneumonia, other serious conditions such as meningitis or endocarditis can occur ^2^.

### Available vaccines, their indications and implications

Currently, there are three vaccines available for clinical use:

- 23-valent polysaccharide vaccine (PPSV23).
- 13-valent conjugate vaccine (PCV13).
- 20-valent conjugate vaccine (PCV20): recently approved by the United States Food and Drug Administration (FDA) and available in few countries (29).

**Figure 2:**
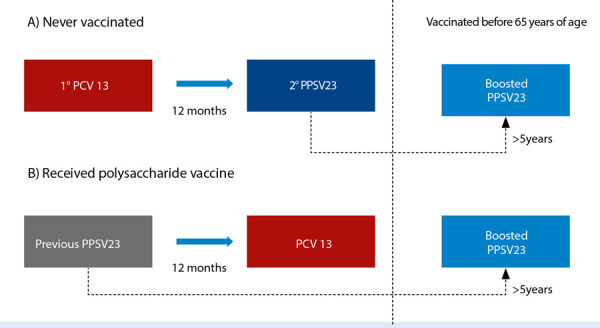
Sequential vaccination schedule with pneumococcal vaccines in high-risk individuals.

The three vaccines are composed of purified surface polysaccharides from different pneumococcal serotypes and the number in their name indicates the number of serotypes they contain. The serotypes contained in the PCV13 and PPSV23 vaccines are complementary, and while their immunogenicity differs, sequential vaccination with both has been shown to be superior to the use of each vaccine separately ^2^^,^^19^^,^^30^^,^^31^. The vaccination indications are very similar to those of the influenza vaccine (Table 2). If possible, initial vaccination with PCV13 is preferred, followed by PPSV23, 12 months after the first dose (Figure 2, scheme A). In individuals who have previously received one or more doses of PPSV23, the schedule should be completed with PCV13 at least 12 months after the last dose administered (Figure 2, scheme B).

**Table 2:** Similarities and differences in the indications for pneumococcal and influenza vaccination from a cardiological perspective

Indication for both vaccines	- Heart failure^*^, coronary artery disease with or without revascularization, valve replacement, moderate or severe valve disease, pulmonary hypertension, heart transplantation. - Congenital heart disease of any severity. - Individuals aged 65 years and older, regardless of comorbidities. - Comorbidities: mostly the same as for influenza vaccine (see below).
Exclusive indications for pneumococcal vaccine	- Active smoking of at least 15 pack-years, or former smokers of at least 10 pack-years if they quit within the last 10 years ^29^. - History of invasive pneumococcal disease. - Alcoholism. - Cochlear implants and cerebrospinal fluid leaks.
Exclusive indications for influenza vaccine	- Healthcare personnel - Obese individuals with a body mass index ≥40 kg/m²

*Regardless of ejection fraction

PPSV23: 23-valent polysaccharide vaccine, PCV 13: 13-valent conjugate vaccine.

**Figure 3:**
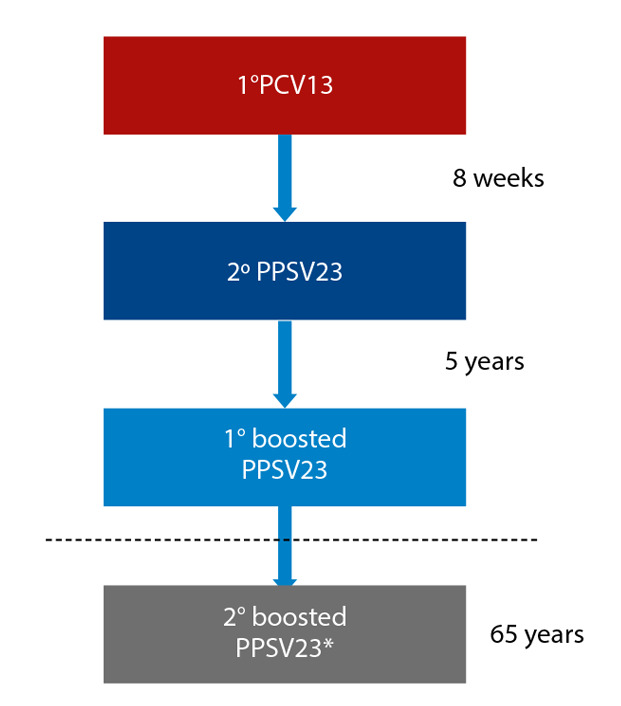
Pneumococcal vaccination schedule in individuals at very high risk for invasive pneumococcal infection, such as heart (or any organ) transplant recipients.

For individuals vaccinated due to risk factors or comorbidities, a booster dose of PPSV23 should be administered after turning 65 years old, and when 5 or more years have elapsed since the last dose of this vaccine. Patients at very high risk for invasive pneumococcal disease, such as heart transplant recipients, due to the lower immune response induced by the PPSV23 vaccine, should receive an additional dose of this vaccine (Figure 3). With the arrival of the PCV20 vaccine, it is expected that this schedule will be simplified, and that individuals at risk will only need to receive a single dose of this vaccine ^19^.

PPSV23: 23-valent polysaccharide vaccine, PCV13: 13-valent conjugate vaccine.

### Reduction of cardiovascular events with the pneumococcal vaccine

There are no clinical trials available that have assessed the impact of this intervention on cardiovascular events. A meta-analysis that included seven observational studies with 163,756 participants showed that in adults with established cardiovascular disease or high cardiovascular risk, pneumococcal vaccination reduced all-cause mortality (HR = 0.78, 95% CI: 0.73-0.83; p <0.001), with low heterogeneity among studies (I^2^ = 32%) ^32^.

### Practical aspects of pneumococcal vaccination

- It can be administered simultaneously with the influenza vaccine and other vaccines recommended for adults (2,19).
- Precautions in individuals on anticoagulants are similar to those used for the influenza vaccine.
- As with influenza vaccine, mild infectious conditions do not represent a contraindication for vaccination ^(^^2^.
- Similar to the influenza vaccine, the only contraindication for the pneumococcal vaccine is a history of anaphylactic reaction.

## Herpes Zoster (shingles) vaccine

Herpes zoster (HZ) is a neurocutaneous disease caused by the reactivation of the primary infection by the varicella-zoster virus (VZV) ^33^. Chickenpox is a highly prevalent condition worldwide; thus, it is estimated that more than 90% of the world’s population over the age of 50 has experienced it at some point, either symptomatically or asymptomatically. Immunity against HZ is acquired for the first time innately upon contracting the infection (primary infection), and subsequent exposures maintain this immunity ^33^ (Figure 3). However, immunity begins to decline with age, due to immunosenescence, or when individuals develop other pathologies or comorbidities that alter it ^22^^,^^34^. The process of immunosenescence begins around the age of 50, where the incidence of the disease is around 2-4.6 cases per 1000 person-years, and increases to 10-12.8 cases per 1000 person-years in octogenarians. Thus, globally, it has been estimated that up to 1 in every 3 individuals between the ages of 50 and 90 will experience an episode of HZ ^34^. However, there are conditions that increase the risk of developing HZ at younger ages (Table 3).

**Table 3:** Comorbidities and risk factors for herpes zoster reactivation in adults

Comorbidities
- Heart failure in functional class III-IV
- Pulmonary hypertension
- Diabetes
- Chronic obstructive pulmonary disease (COPD) and bronchial asthma
- Chronic kidney disease, especially patients on dialysis
- Cardiac transplantation
- Cancer patients
- Human Immunodeficiency Virus (HIV)
- Other comorbidities: depression, chronic liver disease, alcoholism, connective tissue diseases, bone marrow or other organ transplantation.
Immunosuppression and biologic drugs
- Pharmacological: chronic use of corticosteroids, use of monoclonal antibodies, etc.
- Non-pharmacological: splenectomized patients.

### Herpes Zoster (Shingles) and Cardiovascular Events

**Figure 4:**
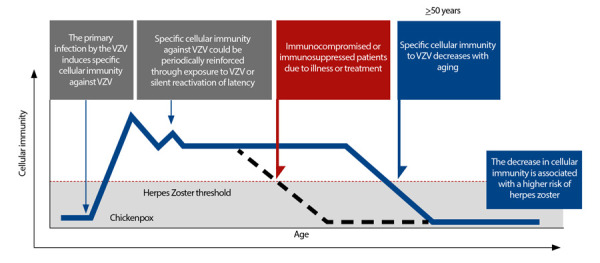
Schematic description of the acquisition of natural immunity against varicella-zoster virus (VZV), and the loss of this immunity with age or the presence of comorbidities (Adapted from reference (33)).

Beyond the inconveniences associated with the acute development of HZ, the main problem of this condition relates to its long-term complications. Among these, the principal and most frequent is postherpetic neuralgia, which can affect up to 30% of individuals with HZ ^35^. The pain of postherpetic neuralgia is often severe, sometimes disabling, and can persist from weeks or months to years after the HZ episode. It has also been observed that after an episode of HZ, cardiovascular and cerebrovascular events increase, as well as ophthalmic and neurological complications ^34^^-^^36^ (Figure 4).

VVZ: virus de la varicela-zóster.

As with other infections, when VZV reactivates it generates an inflammatory phenomenon that can trigger endothelial dysfunction, thus favoring and explaining the occurrence of vascular complications ^36^. Among these, HZ has been associated with:

- Stroke: the increased risk during the first week post-HZ showed an adjusted incidence of 2.37 (95% CI: 2.17-2.59; p<0.05). This risk gradually decreased in the following weeks, reaching 1.55 (95% CI: 1.46-1.65; p<0.05) during the fourth week post-event (37). However, some publications indicate that the risk remains elevated for approximately 1 year after an HZ event ^36^^,^^37^. Additionally, younger patients and those with ophthalmic HZ appear to have a higher risk of developing stroke ^(^^38^.

- Acute myocardial infarction: during the first week following an HZ the adjusted incidence was 1.68 (95% CI: 1.47-1.92; p<0.05), a risk that gradually decreased after 4 weeks to 1.34 (95% CI: 0.98-1.82) ^37^^,^^39^.

- Heart Failure: in a registry from South Korea, individuals who experienced severe cases of HZ requiring hospitalization had an increased risk of heart failure (HR = 2.03, 95% CI: 1.62-2.56; p<0.05) ^39^.

As a result, some studies have explored the role of prescribing antiviral drugs to reduce the risk of ischemic events following the development of HZ, a strategy that has not shown effectiveness so far ^37^.

### Vaccines to prevent HZ (Shingles)

In 2006, the first vaccine against HZ (Zostavax®) was approved, which proved to be an intervention with a good safety profile. However, its main limitations were:

- It is a vaccine with live attenuated virus, which means it cannot be administered to severely immunosuppressed populations (such as transplant recipients), which simultaneously constitutes a condition with a high risk of developing HZ.
- Its efficacy was moderate (51.3% reduction of HZ), with lower response in elderly people.

In 2017, the FDA approved the recombinant HZ vaccine (Shingrix®), which contains an antigen and an adjuvant, overcoming the problems of its predecessor: since it does not contain live attenuated virus, it has no restrictions due to comorbidities leading to severe immunosuppression. Additionally, the recombinant vaccine showed very high efficacy in preventing the development HZ in individuals over 50 years old (HR = 97.2%, 95% CI: 93.7%-99.0%; p<0.001), being also highly effective in those over 70 years old (HR = 89.8%, 95% CI: 84.2%-93.7%; p<0.001). Similarly, the efficacy in preventing postherpetic neuralgia was very high in both age groups: 91.2% (95% CI: 75.9%-97.7%; p<0.05) in individuals over 50 years old, and 88.8% (95% CI: 68.7%-97.1%; p<0.05) in those over 70 years old.

Recently, the 10-year follow-up data of patients included in the initial studies of the vaccine (ZOE-50 and ZOE-70) were released ^40^^,^^41^, demonstrating persistent serological efficacy (both cellular and humoral immunity) as well as clinical efficacy ^42^. Regarding AEs of the recombinant vaccine, these were mostly local, mild, and self-limited. Thus, 79.1% of the participants reported pain, 39.2% erythema, and 26.3% swelling at the injection site. Systemic symptoms were slightly more frequent in younger patients: myalgia was present in 46.3% of those over 50 years old and 31.2% of those over 70 years old, and the same was true for fever (21.5% and 12.3%, respectively). In all cases, these symptoms were transient and self-limited ^40^^,^^41^.

### Reduction of cardiovascular events with the HZ vaccine

A cohort study based on data from United States Medicare beneficiaries included 1,603,406 individuals who were vaccinated with the live attenuated virus vaccine, comparing them with the same number of individuals in the Medicare program who had not been vaccinated. The participants selected as “controls” were “matched” using a propensity score matching model. Participants had a mean follow-up of 5.1 years, and vaccinated individuals showed lower risk of total stroke (HR = 0.84, 95% CI: 0.83-0.85; p<0.001), ischemic stroke (HR = 0.83, 95% CI: 0.82-0.84; p<0.001) and hemorrhagic stroke (HR = 0.88, 95% CI: 0.85-0.91; p<0.001) ^43^.

**Figure 5:**
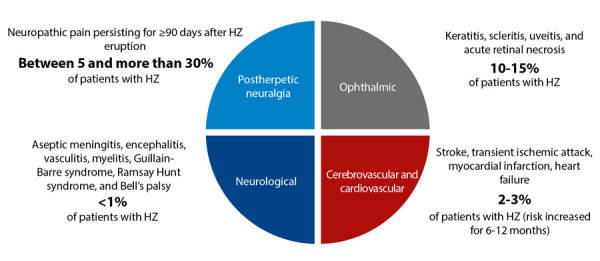
Schematic of complications following a herpes-zoster event and their approximate frequency in immunocompetent adults (Adapted from reference: 35)

HZ: herpes-zoster - PHN: postherpetic neuralgia.

Another study using the TriNetX database analyzed the impact of the recombinant vaccine on the occurrence of cardiovascular events ^44^. They conducted a similar analysis to the previous one, comparing 7,657 individuals who received two doses of the recombinant vaccine with participants from the same database who were not vaccinated, using a propensity score matching model. With an mean follow-up of 3 years, vaccinated participants had a lower RR of myocardial infarction (0.73, 95% CI: 0.55-0.96; p<0.05) and all-cause mortality (RR = 0.84, 95% CI: 0.74-0.95; p<0.05).

### Vaccination schedule and practical aspects of the HZ vaccine

The recombinant vaccine is indicated for individuals aged 50 years and older, regardless of the presence of comorbidities, and for younger individuals with risk factors ^19^^)^ (Table 3). The vaccination schedule is very simple, as it involves administering the first dose at the time of the consultation, followed by a booster dose 2-6 months after the first dose. The vaccine is administered intramuscularly.

### Some practical aspects to consider are:

- Serological testing for VZV prior to immunization is not required (nor recommended).
- It can be administered simultaneously (or a few days before or after) with influenza, conjugate and polysaccharide pneumococcal vaccines, double adults and COVID-19 vaccines.
- The only contraindication to the vaccine is hypersensitivity to any of its components.

## COVID-19 vaccine

The SARS-CoV-2 virus infection (COVID-19) was declared a pandemic in March 2020 ^19^. In addition to severe respiratory infections, COVID-19 has resulted in serious and even fatal cardiovascular complications ^45^^,^^46^. The introduction of COVID-19 vaccines has dramatically reduced the occurrence of severe disease, and observational studies have documented a lower rate of major cardiovascular events associated with this infection ^47^. Therefore, it is currently recommended that all adults receive annual immunization against COVID-19, and that individuals at increased risk receive booster doses every 6 months ^19^. However, due to the dynamic nature of this disease, it is not possible to determine whether these recommendations will remain in place over time.

## Respiratory syncytial virus vaccine

Respiratory Syncytial Virus (RSV) is a single-stranded RNA virus, highly contagious and distributed worldwide, which causes hundreds of thousands of infections, leading to a high number of hospitalizations and deaths ^19^^,^^48^. The most susceptible subgroups are infants and adults with comorbidities, mainly cardiovascular, respiratory, metabolic, or immunological disorders.

Similar to other conditions, RSV infections have been documented to be associated with *de novo* cardiovascular events and exacerbation of pre-existing conditions ^48^. Due to this, the FDA recently approved two vaccines for clinical use in individuals aged 60 years and older: Arexvy® and Abrysvo® ^19^. These interventions have shown a good safety profile, with very high effectiveness in elderly individuals with cardiovascular comorbidities ^48^^,^^49^. These observations reinforce the idea that individuals with heart disease are a group that will particularly benefit from this intervention.

## Role of the cardiologist in adult immunizations

Low vaccination rates in adults are a common issue worldwide ^1^^,^^12^^,^^13^. Although there are multiple factors that influence this, most series indicate that healthcare professionals are the main determinants of this reality ^1^^,^^50^ (Table 4). It has been demonstrated that when the treating physician takes a proactive approach in recommending vaccination, immunization rates are significantly higher ^50^.

**Table 4:** Attitude of individuals towards vaccination recommendations and reasons for the lack of utilization of this intervention

They would get vaccinated if their doctor recommends it	85%
Effective vaccination rate among survey participants	56% - 61%
Reasons for not having been vaccinated		
“The doctor did not prescribe it for me”	57%
“If I feel healthy, I don’t need it”	61%
Fear of adverse effects	40%
Economic difficulties for access	17%

(Adapted from (50))

The prevention of cardiovascular events is one of the central axes of cardiology, and the improvements achieved in this area have made it possible to increase life expectancy and quality of life. Vaccines are undoubtedly another tool to reduce residual cardiovascular risk. The challenge lies in developing strategies to achieve and maintain high vaccination rates, transforming ourselves into facilitators rather than barriers to immunization. Checklists, electronic reminders in medical records, electronic messaging to patients, and community education about the role of vaccines in cardiovascular prevention are some of the tools that can be used for this purpose

## Conclusion

There is a close relationship between infections affecting various organs, major cardiovascular and cerebrovascular events, and mortality. In the case of respiratory infections, concomitant hypoxemia increases this association. Simultaneously, vaccination has proven to be a safe and cost-effective strategy to reduce these events. However, vaccination rates in adults are extremely low, even in populations at very high cardiovascular risk.

Given all of the above, it is imperative to adopt immunizations as an indispensable strategy to reduce overall cardiovascular risk and achieve effective cardiovascular prevention.

## References

[1] Garcia-Zamora S, Sosa Liprandi MI, Picco JM, Matta MG, Villarreal R, Pulido L (2020). Immunizations in adults with cardiovascular disease. Summary of the Consensus of the Argentine Cardiology Society. Medicina (B Aires).

[2] Roth GA, Mensah GA, Fuster V (2020). The Global Burden of Cardiovascular Diseases and Risks A Compass for Global Action. J Am Coll Cardiol.

[3] Dugani S, Gaziano TA (2016). 25 by 25 Achieving Global Reduction in Cardiovascular Mortality. Curr Cardiol Rep.

[4] Everett BM (2019). Residual Inflammatory Risk A Common and Important Risk Factor for Recurrent Cardiovascular Events. J Am Coll Cardiol.

[5] Mattila KJ (1989). Viral and bacterial infections in patients with acute myocardial infarction. J Intern Med.

[6] Smeeth L, Thomas SL, Hall AJ, Hubbard R, Farrington P, Vallance P (2004). Risk of myocardial infarction and stroke after acute infection or vaccination. N Engl J Med.

[7] Corrales-Medina VF, Musher DM, Wells GA, Chirinos JA, Chen L, Fine MJ (2012). Cardiac complications in patients with community-acquired pneumonia incidence, timing, risk factors, and association with short-term mortality. Circulation.

[8] Sethi NJ, Safi S, Korang SK, Hrobjartsson A, Skoog M, Gluud C (2021). Antibiotics for secondary prevention of coronary heart disease. Cochrane Database Syst Rev.

[9] Warren-Gash C, Bhaskaran K, Hayward A, Leung GM, Lo SV, Wong CM (2011). Circulating influenza virus, climatic factors, and acute myocardial infarction a time series study in England and Wales and Hong Kong. J Infect Dis.

[10] Warren-Gash C, Blackburn R, Whitaker H, McMenamin J, Hayward AC (2018). Laboratory-confirmed respiratory infections as triggers for acute myocardial infarction and stroke a self-controlled case series analysis of national linked datasets from Scotland. Eur Respir J.

[11] Vardeny O, Claggett B, Udell JA, Packer M, Zile M, Rouleau J (2016). Influenza Vaccination in Patients with Chronic Heart Failure The PARADIGM-HF Trial. JACC Heart Fail.

[12] Matta MG, Pulido L, Herrera-Paz JJ, Picco JM, Wolff S, Tse G (2023). Influenza and pneumococcal vaccine prescription for adults during COVID-19 first wave in three regions of Argentina. Vaccine.

[13] Sosa Liprandi A, Zaidel EJ, López Santi R, Araujo JJ, Banos González MA, Busso JM (2021). Influenza and Pneumococcal Vaccination in Non-Infected Cardiometabolic Patients from the Americas during the COVID-19 Pandemic A Sub-Analysis of the CorCOVID-LATAM Study. Vaccines (Basel).

[14] Gurfinkel EP, R Leon de la Fuente, Mendiz O, Mautner B (2004). Flu vaccination in acute coronary syndromes and planned percutaneous coronary interventions (FLUVACS) Study. Eur Heart J.

[15] Udell JA, Zawi R, Bhatt DL, Keshtkar-Jahromi M, Gaughran F, Phrommintikul A (2013). Association between influenza vaccination and cardiovascular outcomes in high-risk patients a meta-analysis. JAMA.

[16] Yedlapati SH, Khan SU, Talluri S, Lone AN, Khan MZ, Khan MS (2021). Effects of Influenza Vaccine on Mortality and Cardiovascular Outcomes in Patients with Cardiovascular Disease A Systematic Review and Meta-Analysis. J Am Heart Assoc.

[17] Frobert O, Gotberg M, Erlinge D, Akhtar Z, Christiansen EH, MacIntyre CR (2021). Influenza Vaccination After Myocardial Infarction A Randomized, Double-Blind, Placebo-Controlled, Multicenter Trial. Circulation.

[18] Grohskopf LA, Blanton LH, Ferdinands JM, Chung JR, Broder KR, Talbot HK (2022). Prevention and Control of Seasonal Influenza with Vaccines Recommendations of the Advisory Committee on Immunization Practices - United States, 2022-23 Influenza Season. MMWR Recomm Rep.

[19] Luna CM, Pulido L, Rizzo O, Gauna ML, Chirino A, Videla AJ (2023). Recomendaciones actualizadas para la vacunación de adultos con enfermedades respiratorias. Asociación Argentina de Medicina Respiratoria. Medicina (B Aires).

[20] Tamerius J, Nelson MI, Zhou SZ, Viboud C, Miller MA, Alonso WJ (2011). Global influenza seasonality reconciling patterns across temperate and tropical regions. Environ Health Perspect.

[21] Tricco AC, Chit A, Soobiah C, Hallett D, Meier G, Chen MH (2013). Comparing influenza vaccine efficacy against mismatched and matched strains a systematic review and meta-analysis. BMC Med.

[22] Crooke SN, Ovsyannikova IG, Poland GA, Kennedy RB (2019). Immunosenescence and human vaccine immune responses. Immun Ageing.

[23] Gravenstein S, McConeghy KW, Saade E, Davidson HE, Canaday DH, Han L (2021). Adjuvanted Influenza Vaccine and Influenza Outbreaks in US Nursing Homes Results from a Pragmatic Cluster-Randomized Clinical Trial. Clin Infect Dis.

[24] Johansen ND, Modin D, Nealon J, Samson S, Salamand C, Larsen CS (2022). Feasibility of randomizing Danish citizens aged 65-79 years to high-dose quadrivalent influenza vaccine vs standard-dose quadrivalent influenza vaccine in a pragmatic registry-based setting: rationale and design of the DANFLU-1 Trial. Pilot Feasibility Stud.

[25] Stein AN, Mills C, McGovern I, Dean A, Bogdanov A, Sullivan S, Haag M Superior Effectiveness of Cell-Based Versus Egg-Based Quadrivalent Influenza Vaccines Against Outpatient Test-Confirmed Influenza Over Three Consecutive Seasons in the United States (V130_67RWE).

[26] Vardeny O, Kim K, Udell JA, Joseph J, Desai AS, Farkouh ME (2021). Effect of High-Dose Trivalent vs Standard-Dose Quadrivalent Influenza Vaccine on Mortality or Cardiopulmonary Hospitalization in Patients With High-risk Cardiovascular Disease A Randomized Clinical Trial. JAMA.

[27] McNeil MM, Weintraub ES, Duffy J, Sukumaran L, Jacobsen SJ, Klein NP (2016). Risk of anaphylaxis after vaccination in children and adults. J Allergy Clin Immunol.

[28] Said MA, Johnson HL, Nonyane BA, Deloria-Knoll M, OBrien KL, Team AAPBS (2013). Estimating the burden of pneumococcal pneumonia among adults a systematic review and meta-analysis of diagnostic techniques. PLoS One.

[29] Essink B, Sabharwal C, Cannon K, Frenck R, Lal H, Xu X (2022). Pivotal Phase 3 Randomized Clinical Trial of the Safety, Tolerability, and Immunogenicity of 20-Valent Pneumococcal Conjugate Vaccine in Adults Aged &gt;/=18 Years. Clin Infect Dis.

[30] Kobayashi M, Bennett NM, Gierke R, Almendares O, Moore MR, Whitney CG (2015). Intervals Between PCV13 and PPSV23 Vaccines Recommendations of the Advisory Committee on Immunization Practices (ACIP). MMWR Morb Mortal Wkly Rep.

[31] Jiménez Ruiz CA, Buljubasich D, Sansores R, Riesco Miranda JA, Guerreros Benavides A, Luhning S (2015). SEPAR-ALAT Consensus Document on Antipneumoccal Vaccination in Smokers. Arch Bronconeumol.

[32] Marques Antunes M, Duarte GS, Brito D, Borges M, Costa J, Ferreira JJ (2021). Pneumococcal vaccination in adults at very high risk or with established cardiovascular disease systematic review and meta-analysis. Eur Heart J Qual Care Clin Outcomes.

[33] Arvin A (2005). Aging, immunity, and the varicella-zoster virus. N Engl J Med.

[34] Curran D, Callegaro A, Fahrbach K, Neupane B, Vroling H, van Oorschot D (2022). Meta-Regression of Herpes Zoster Incidence Worldwide. Infect Dis Ther.

[35] Sundstrom K, Weibull CE, Soderberg-Lofdal K, Bergstrom T, Sparen P, Arnheim-Dahlstrom L (2015). Incidence of herpes zoster and associated events including stroke--a population-based cohort study. BMC Infect Dis.

[36] Addario A, Celarier T, Bongue B, Barth N, Gavazzi G, Botelho-Nevers E (2023). Impact of influenza, herpes zoster, and pneumococcal vaccinations on the incidence of cardiovascular events in subjects aged over 65 years a systematic review. Geroscience.

[37] Warren-Gash C (2018). Herpes Zoster Epidemiological Links With Stroke and Myocardial Infarction. J Infect Dis.

[38] Erskine N, Tran H, Levin L, Ulbricht C, Fingeroth J, Kiefe C (2017). A systematic review and meta-analysis on herpes zoster and the risk of cardiac and cerebrovascular events. PLoS One.

[39] Seo HM, Cha MJ, Han JH, Han K, Park SH, Bang CH (2018). Reciprocal relationship between herpes zoster and cardiovascular diseases A nationwide population-based case-control study in Korea. J Dermatol.

[40] Lal H, Cunningham AL, Godeaux O, Chlibek R, Diez-Domingo J, Hwang SJ (2015). Efficacy of an adjuvanted herpes zoster subunit vaccine in older adults. N Engl J Med.

[41] Cunningham AL, Lal H, Kovac M, Chlibek R, Hwang SJ, Diez-Domingo J (2016). Efficacy of the Herpes Zoster Subunit Vaccine in Adults 70 Years of Age or Older. N Engl J Med.

[42] Strezova A, Diez-Domingo J, Al Shawafi K, Tinoco JC, Shi M, Pirrotta P (2022). Long-term Protection Against Herpes Zoster by the Adjuvanted Recombinant Zoster Vaccine Interim Efficacy, Immunogenicity, and Safety Results up to 10 Years After Initial Vaccination. Open Forum Infect Dis.

[43] Yang Q, Chang A, Tong X, Merritt R (2021). Herpes Zoster Vaccine Live and Risk of Stroke Among Medicare Beneficiaries A Population-Based Cohort Study. Stroke.

[44] Helm MF, Khoury PA, Pakchanian H, Raiker R, Maczuga S, Foulke GT (2023). Recombinant Zoster Vaccine Reduces 3-Year Cardiovascular Risk Insights from a Multi-Centered Database. J Drugs Dermatol.

[45] Garcia-Zamora S, Lee S, Haseeb S, Bazoukis G, Tse G, Alvarez-Garcia J (2021). Arrhythmias and electrocardiographic findings in Coronavirus disease 2019: A systematic review and meta-analysis. Pacing Clin Electrophysiol.

[46] Garcia-Zamora S, Picco JM, Lepori AJ, Galello MI, Saad AK, Ayon M (2023). Abnormal echocardiographic findings after COVID-19 infection a multicenter registry. Int J Cardiovasc Imaging.

[47] Jiang J, Chan L, Kauffman J, Narula J, Charney AW, Oh W (2023). Impact of Vaccination on Major Adverse Cardiovascular Events in Patients With COVID-19 Infection. J Am Coll Cardiol.

[48] Ivey KS, Edwards KM, Talbot HK (2018). Respiratory Syncytial Virus and Associations with Cardiovascular Disease in Adults. J Am Coll Cardiol.

[49] Feldman RG, Antonelli-Incalzi R, Steenackers K, Lee DG, Papi A, Ison MG (2024). Respiratory Syncytial Virus Prefusion F Protein Vaccine Is Efficacious in Older Adults with Underlying Medical Conditions. Clin Infect Dis.

[50] Johnson DR, Nichol KL, Lipczynski K (2008). Barriers to adult immunization. Am J Med.

